# Integrated Community Case Management Utilization Status and Associated Factors Among Caretakers of Sick Children Under the Age of 5 Years in West Shewa, Ethiopia

**DOI:** 10.3389/fpubh.2022.929764

**Published:** 2022-07-20

**Authors:** Lemessa Negeri Debel, Fikadu Tadesse Nigusso

**Affiliations:** ^1^Department of HIV and TB Research Directorate, Ethiopian Public Health Institute, Addis Ababa, Ethiopia; ^2^School-Based Programme Unit, World Food Programme, Addis Ababa, Ethiopia

**Keywords:** integrated community case management (ICCM), utilization, associated factors, under-five children, demand creation

## Abstract

**Objective:**

To assess the utilization status and associated factors of integrated community case management (ICCM) of caretakers with <5 years of sick children.

**Methods:**

Community-based cross-sectional study was employed with caretakers whose child was sick in the last 3 months before data collection. Bivariate and multivariable logistic regression analyses were employed.

**Results:**

About 624 respondents participated in the study; 325 (52.1%) utilized integrated community case management. Caring for children between the ages 24–36 months old, (AOR = 1.26, 95%CI: 0.23, 0.90); women health development army (WHDA) training, (AOR = 5.76, 95%CI: 3.57, 9.30); certified as model family, (AOR = 3.98, 95%CI: 2.45, 6.46); perceived severity, (AOR = 5.29, 95%CI: 2.64, 10.60); awareness of danger sign, (AOR = 2.76, 95%CI: 1.69, 4.50), and awareness of ICCM, (AOR = 5.42, 95%CI: 1.67, 17.58) were associated with ICCM utilization.

**Conclusion:**

This study revealed that age of the child, caretakers' awareness of ICCM, awareness of danger signs, illness severity, women's health developmental army training, and graduation as a model family were associated with ICCM utilization. Therefore, it is recommended that promote health education using community-level intervention modalities focusing on common childhood illness symptoms, danger signs, severity, and care-seeking behavior.

## Introduction

With global communities' commitments, a dramatic reduction was registered in child mortality over the last 30 years; under-five mortality has dropped by almost 38 deaths per 1,000 live births in 2019 from 93 in 1990, albeit the global burden of child death under theee age of 5 remains immense (1). In 2020 alone, 5 million children under 5 years of age died mostly of preventable or treatable causes (2). Nearly half of these deaths occurred in sub-Saharan Africa with an average under-five ality rate of 76 deaths per 1,000 live births equating to one child in 13 dying before reaching age five despite the availability of effective, relatively low-cost interventions to prevent and treat the main causes of child mortality (1). Integrated community case management of childhood illness (ICCM) is one such effective, low-cost strategy to train, support, and supply health workers to provide clinical care for common childhood illnesses in communities (3).

Integrated community case management is the child survival intervention strategy designed by WHO and UNICEF to address and reduce child morbidity and mortality through health promotion, prevention, and curative service by engaging the community in the process. The strategy includes the health care provider capacity building through training in management of common childhood illnesses, health facility equipment, and capacity building of community to identify their child's illness by the three common symptoms (diarrhea/vomiting, cough, and fever), and seek treatment and/or care timely, and give care to the child at home (4). ICCM principally brings treatment services closer to home, particularly to those with poor access to health facilities, and advocated for low- and middle-income countries in the 2000s (5). Both WHO and UNICEF recommend ICCM as a strategy to improve equity in access to health care. Consequently, to increase coverage of life-saving interventions, countries have increasingly implemented ICCM through community-based health workers.

In the year 2019, an estimated 5.2 million under the age of 5 years died from preventable and treatable diseases such as preterm birth complications, birth asphyxia/trauma, pneumonia, congenital anomalies, diarrhea, and malaria (6), all of which can be prevented or treated with access to simple, affordable interventions including immunization, adequate nutrition, safe water, and food and quality care by a trained health provider using integrated community case management strategies. There is evidence that ICCM has improved access to appropriate case management, which leads to earlier treatment, fewer severe cases, improved recovery, fewer referrals, and a reduced burden on health service deliveries (4). Similarly, studies have shown that ICCM can increase coverage of appropriate treatment for childhood illnesses among the poor and can lead to substantial reductions in child mortality (7–10).

Cognizant of the challenge and to reduce child mortality in children <5 years old, the government of Ethiopia has also made a series of efforts by including ICCM into health policy and scaleup ICCM implementation. The MOH adopted ICCM principles in their policy which was later rolled out as integrated community case management, a decentralized community level implemented approach using community health workers, primarily by Health Extension Workers (HEWs) (11). Health Extension Workers accomplish the preventive and curative service of this program in the community through counseling and health education to create awareness and demand for care-seeking utilization among caretakers for their child's illness during outreach and home-to-home visit (12). Accordingly, health extension workers provide care and/ or treatment for the ill child within the communities as recognized by three common symptoms: diarrhea and/ or vomiting, cough, and fever. Thus, the care is not delayed, mostly within 24 h of illness recognized, and the child is taken to an appropriate health facility or trained provider (4). Despite such efforts, the ICCM demand creation and the level of ICCM utilization among sick children under the age of 5 are low in the country (13).

The improvement attributed to ICCM implementation, and the possible factors related to caretakers' demand, and utilization of the ICCM program are not well-studied in the study area. Existing studies focused on health facilities' performance rather than on the perspective, awareness, and care-seeking practice of caretakers (8, 14). As Ethiopia is one of the countries committed to realizing the SDG goal of limiting child mortality in children under 5 years to 20/100 live births by 2030 (15, 16), it is highly important to know the improvement of caretakers' utilization of care-seeking made and the factors associated with it. Therefore, the purpose of the study was to assess the utilization status and associated factors of integrated community case management of caretakers of sick children <5 years of age in Dandi woreda, West Shewa Zone, Ethiopia. The finding of this study is helpful for both policymakers and health practitioners to strengthen and/or redesign resource-efficient and effective community-based strategies paramount to resource-limited settings such as Ethiopia that significantly reduce child morbidity and mortality.

## Methods

### Study Setting and Period

The study was conducted in Dandi woreda, West Shewa Zone, Oromia Regional State, Ethiopia between January and February 2017. Dandi is bordered on the south by the South-west Shewa Zone, on the west by Ambo, and located 112 km from the capital Addis Ababa. The district has 48 rural kebele with nearly 224,860 population: 36,944 children were under 5 years of age. During the study period, eight health centers and 48 rural health posts were providing integrated community case management since 2011.

### The Sample Population

The source population for this study was all caretakers of children of <5 years in the Dandi district while all caretakers of <5 years old caretakers in Dandi woreda were the study populations.

### Study Design

The community-based cross-sectional study design was conducted among caretakers of sick children who were <5 years of Dandi District, West Shewa, Oromia Regional State, Ethiopia.

### Sample Size Determination

The sample size for the study was calculated using single population proportion formula considering 43.6% prevalence of integrated community case management utilization of caretakers in the study conducted in Shashigo Hadiya Zone, Southern Ethiopia (17) with 5% marginal error at a 95% confidence level. Accordingly, 416 samples were studied. The sample size was computed as $n=\left(Z\propto \frac{1}{2}\right)$2$*\frac{p\left(1-p\right)}{2}$, $n=\left(Z\propto \frac{1}{2}\right)$2$*\frac{pq}{\left(d\right)2}$ = (1.9)2*378. Where: *n* = required sample size; *Z*∝/2 = critical value for normal distribution at 95% confidence level which equaled to 1.96 (z value at α = 0.05); *p* = 43.6% as the study conducted at Shashigo of Hadiya Zone, Southern Ethiopia public health facility; d = an absolute precision margin of error 5%. The calculated sample size was 378. To cut the non-response rate, 10% were added to cater to the non-response rate resulting in 416 caretakes of children under 5 years old. To solve the problem with the design effect, the sample was multiplied by 1.5 (18), thus, 624 eligible households with under-five sick children within 2 weeks before the data collection date were taken.

### Sampling Procedure

Multi-stage systematic sampling was employed using the population-district-village-household-individual approach. In the first stage, 10 kebeles were selected by simple random sampling. Following that, eligible samples of caretakers of <5 years old sick children in the last 3 months among each kebele were obtained from the updated family folder at health posts. The samples were proportionally allocated to each kebeles. Then the “K” value was calculated by dividing the number of sick children under the age of five children of each kebele obtained from the updated family folder at health posts by the proportion of samples needed from each kebele that resulted in five on average. Finally, using a systematic random sampling method, data was collected from 624 households containing sick children <5 years oldat k's interval starting from the first household of any end of the kebele selected using the pin pen method. If there were two and above under five ill children in the house, the youngest child was taken while the latest health problem was respected if the child got ill for more than one time.

### Data Collection

The structured questionnaires were used for the study. The questionnaires were contextually developed based on the study objective and study, and existing national and global scientific evidence (14, 15). The data was collected by university graduates from each kebele or in the district. The validity and reliability of the data collection tool were improved by conducting a pilot test among kebeles not included in the study.

### Operational Definition

- Integrated community case management (ICCM) is the strategy that enables the assessment, classification, and management of childhood illnesses in an integrated manner at the health post level (4). And also, for this study, integrated community case management services utilization means receiving packages of health care services for the child's problems as per the ICCM guidelines reported by the mother or caregiver.
- Danger signs are signs of serious illness that are seen in children aged 2 months up to 5 years and will need immediate action to save the life of the child (19).
- Certified family models are those households that are trained in HEP packages, implement these packages after the training, and can influence their neighbors to adopt the same practices.
- Women's health development army represents a systematic, organized, and collaborative movement through active participatory learning and actions to improve health.
- Integrated community case management utilization refers to mothers' or caretakers' responses as sought advice or treatment at a health post or a health extension worker or health center/hospital or health worker after recognizing her child's illness with the three symptoms of diarrhea, and/or cough, and/or fever without delay (in 24 h of recognition of the illness).
- Perception of the caretaker toward the severity of the illness: defined as the caretakers' reason/ response to seek advice or treatment for her child's illness and for the question of why they did/didn't utilize seeking advice or treatment from health extension workers/health post and/or health workers/ health center, respectively.

### Data Quality and Analysis

The data were checked for completeness, and consistencies during and after each day of data collection, before coding and entering into EP INFO 7.0; kept in a locked cabinet and only the researchers accessed the data. Following that data was checked and cleaned using excel, and finally, imported to and analyzed using the IBM statistical package for the social sciences (SPSS) for windows version 20.0. Binary logistic regression analysis was done to obtain statistical associations. For controlling of confounding effect and to appropriately determine associated factors of integrated community case management service utilization, a multivariate logistic regression analysis was carried out. The strength of statistical association was measured by adjusted odds ratios and 95% confidence intervals. Statistical significance was declared at *p* < 0.05.

## Results

### Socio-Demographic Characteristics

A total of 624 respondents participated in the study. Five hundred fifty-four (88.8%) were mothers of the children and 70 (11.2%) were non-mother caretakers; about 334 (53.5%) were female caretakers. The mean age of sick children was 25 months, while the mean age of caretakers was 30 years old. In terms of ethnic group mix, 558 (89.4%) were Oromos, 52 (8.3%) Amhara, and 14 (2.2%) Gurage. A total of 508 (81.4%) were married, 44 (7.1%) were divorced, and 51 (8.2%) were widowed. Almost half, 315 (50%) of the caretakers were not attended formal education. Table 1 presents the socio-demographic characteristics in terms of ICCM service delivery in Dandi Woreda.

**Table 1:** Socio-demographic characteristics of the study participants in Dandi Woreda, West Shewa, Ethiopia, 2022.

**Variables**	**Sub-types**	**Frequency**	**Percentage**
		**(*n* = 624)**	
**Socio-demographic characteristics**			
Sex of the child	Male	290	46.5
	Female	334	53.5
Age of the child	<11 months	123	19.7
	12–23 months	194	31.1
	24–35 months	185	29.6
	36–59 months	122	19.6
Care takers age	<24 years	127	20.4
	25–34 years	345	55.3
	35–44 years	136	21.8
	>45 years	16	2.6
Religion	Protestant	172	27.6
	Orthodox	309	49.5
	Muslim	20	3.2
	Wakefata	123	19.7
Marital status	Not married	21	3.4
	Married	508	81.4
	Divorced	44	7.1
	Widowed	51	8.2
Educational status	Not attended	315	50.5
	Primary school	296	47.4
	Secondary & above	13	2.1
Occupation	Housewife	436	69.9
	Merchant	119	19.1
	Govt. employee	6	1.0
	Daily laborer	63	10.1
Monthly income	<500 ETB	464	74.4
	501–1,000 ETB	131	21.0
	1,001–1,500 ETB	19	3.0
	>1,501 ETB	10	1.6

### Care Seeking Behaviors and Health Facility Features

The majority of the study participants, 356 (57%), responded that the ICCM service is available within the district's health facilities while 268 (43%) replied none. The vast majority of the community health care workers (the health extension workers) live out of the assigned kebeles, 68.6%. It was reported by 236 (37.8%) of the respondents that most health facilities are 1 to 2 h far from the local communities. In terms of transportation, about 414 (66%) of caretakers had reported that there was no transportation access to reach the ICCM service center.

Integrated community case management utilization varies with multiple factors associated with study participants. For instance, it varies with the presence of a health facility - among all caretakers whose child was ill, only 325 (52.1%) utilized ICCM by seeking treatment and/or advice at a health facility and/or health worker, while 299 (47.9%) were not. Of all, about 111 (34.15%) received the ICCM service at the health post, 205 (62.46%) in the health center, and 9 (2.77%) in the hospital. In terms of onset of illness, care was sought for 56 (17.2%) on the first date, 200 (61.54%) on the 2nd day, and 69 (21.2%) on the third day after the onset of the illness.

The integrated community case management utilization also differs with the availability of health facilities and health workers. In the health post, about (55.1%) utilized ICCM; about 52.2% where health extension workers (52.2%) were available, and in a place where service is rendered in 60 min and less (61.9%), and those who have transportation access to go to the health center (57.1%) had utilized ICCM than their counterparts.

The reasons for not being sought at health posts include the absence of enough drugs 380 (74.07%); absence of HEW at health posts 325 (63.35%), waiting for a long time 63 (12.28%); and distance from home 8 (1.56%). Analogously the possible reasons mentioned by the caretakers that made them not utilized care for their child's illness at the health center include distance and lack of transportation, service expensiveness, waiting for a long time and health workers were not cooperative. Given such challenges, the respondents admitted the use of private clinics (33.45%), traditional healers (15.05%), drug vendors (8.70%), and religious centers (8.03%) as an alternative care-seeking centers. Table 2 shows the caretakers' ICCM utilization and associated factors.

**Table 2:** Health facility characteristics and integrated community case management (ICCM) service utilization status in Dandi woreda, West Shewa, Ethiopia, 2022.

**Variables**	**Variables sub-types**	**Frequency (*n* = 624)**	**Percent**
Assigned HEWs	Yes	609	97.60
	No	265	42.50
Place of HEWs live	In the kebele	196	31.40
	Out of the kebele	428	68.60
Place where care was received	Public health post	111	34.15
	Public health center	205	62.46
	Public hospital	9	2.77
	Private clinic	100	33.45
	Not in the health facility (traditional healers, drug vendors, religious Centers)	95	31.78
	Not sought at all	104	35.80
Care sought at HF (*n* = 624)	Yes	325	52.10
	No	299	47.90
Time is taken to the health facility	<60 min	139	22.30
	61–120 min	236	37.80
	121–180 min	187	30.00
	>181 min	62	9.90
Transportation to HC	Yes	210	33.70
	No	414	66.30
The time when sought (*n* = 325)	On the 1stday	56	17.20
	Second day	200	61.54
	Third and above	69	21.20
Reasons for not utilizing ICCM service at a private clinic (*n* = 524)	Not severe or resolved by itself	114	57.30
	Distance or lack of transportation	41	20.60
	Expensive cost/Lack of money	24	12.00
	Waiting for a long time /Waiting for permission	11	5.50
	Busy for work	9	4.50
Reasons for not utilization of ICCM service at health post (*n* = 513)	Distance/transportation	8	1.56
	No HEWs/closed HP	325	63.35
	HP has no drug and laboratory	380	74.07
	HEWs have no skill	320	62.38
	Waiting for a long time	63	12.28
	Other (not known as service given)	56	10.92
Reasons for not utilization of ICCM service at the health center (*n* = 419)	Distance or lack of transportation	197	47.00
	To use at HP	109	26.00
	Expensive (Cost)	92	22.00
	Other (no patient friendly service)	21	5.00

### Care-Takers Awareness and Perception of a Child's Illness in the Utilization of Integrated Community Case Management

The utilization of integrated community case management is affected by participants' level of awareness and perception of their child's illness. Their perception varies with the age, marital, and educational status of respondents. Caretakers <24 years old utilized the most in comparison to those >35 years (62.5 *vs*. 48.0%); married respondents seek care two times than widowed (71.4 vs. 35.3%), and respondents with secondary education utilized ICCCM care more than those not attended formal education (72.7 vs. 47.3%).

In terms of clinical symptoms, the participants presented for care-seeking: diarrhea 328 (52.5%), cough 203 (32.5%), fever 196 (31.4%), and vomiting 93 (14.9%). When awareness and perception were studied for the severity of their child's illness, the respondents believe the case is severe when a child has more than one symptom 196 (31.4%); refused to eat/drink (76.8%); illness stays for a long time 142 (28.8%); and high fever 4 (0.4%). About half of the respondents, 312 (50%), did not have awareness of danger signs. Those who had awareness assumed the danger sign when the child is unable to eat or drink 128 (40.9%), fast breathing or granting 94 (30.1%), unable to move 60 (19.1%), and vomiting everything 30 (9.9%). Of those who had awareness of signs of danger 214 (70.9%) utilized care and/or advice at a health facility and/or health care provider. Respondents who had awareness of ICCM utilized integrated community case management more than those who had not (74.0 vs. 26.0%), and respondents who had training in the health developmental army (71.6 vs. 27.3%). Table 3 presents care-takers awareness and perception of child illness concerning ICCM utilization.

**Table 3:** Socio-demographic and caretaker's awareness and perception of child's illness in ICCM utilization in Dandi woreda, West Shewa, Ethiopia, 2022.

**Variables**	**Variable sub-types**	**Frequency (*n* = 624)**	**Percent**	**ICCM utilization**	
				**Yes *n*(%)**	**No *n*(%)**
Sex of the child	Male	290	46.5	145 (50.0)	145 (50.0)
	Female	334	53.5	180 (53.9)	154 (46.1)
Age of the child	<11 months	123	19.7	68 (55.3)	55 (44.7)
	12–23 months	194	31.1	109 (56.2)	85 (43.8)
	24–35 months	185	29.6	80 (43.2)	105 (56.8)
	36–59 months	122	19.6	68 (55.7)	54 (44.3)
Care takers age	<24 years	127	20.4	61 (48.0)	66 (52.0)
	25–34 years	345	55.3	182 (52.8)	163 (47.2)
	35–44 years	136	21.8	72 (52.9)	64 (47.1)
	>45 years	16	2.6	10 (62.5)	69 (37.5)
Religion	Protestant	172	27.6	98 (57.0)	74 (43.0)
	Orthodox	309	49.5	160 (51.8)	149 (48.2)
	Muslim	20	3.2	5 (25.0)	15 (75.0)
	Wakefata	123	19.7	63 (51.2)	60 (48.8)
Marital status	Not married	21	3.4	15 (71.4)	6 (28.6)
	Married	508	81.4	272 (53.5)	236 (46.5)
	Divorced	44	7.1	20 (45.5)	24 (54.5)
	Widowed	51	8.2	20 (35.3)	31 (64.7)
Symptoms	Diarrhea	328	52.5	161 (49.1)	167 (50.9)
	Fever	196	31.4	110 (56.1)	86 (43.9)
	Cough	203	32.5	116 (57.1)	87 (42.9)
Severity of the problem	Not sever	114	18.3	49 (43.0)	65 (57.0)
	Moderate	319	51.1	130 (40.8)	189 (59.2)
	Sever	191	30.6	146 (76.4)	45 (23.6)
Symptoms perceived as sever	More than 1 day	196	31.4	115 (58.7)	81 (41.3)
	Not eat/drink	479	76.8	248 (51.8)	231 (48.2)
	Illness stays long	142	28.8	65 (45.1)	77 (54.9)
	Other (fever)	5	0.7	4 (75.0)	1 (25.0)
Awareness of danger signs	Yes	302	48.4	214 (70.9)	88 (29.1)
	No	322	51.6	111 (34.5)	211 (65.5)
Women health development army training	Yes	339	54.3	251 (74.0)	88 (26.0)
	No	285	45.7	74 (26.0)	211 (74.0)
Certified Model family	Yes	349	55.9	250 (71.6)	99 (28.4)
	No	275	44.1	75 (27.3)	200 (72.7)
Awareness ICCM	Yes	258	41.3	187 (72.5)	71 (27.5)
	No	366	58.7	138 (37.7)	228 (62.3)

### Factors Associated With Integrated Community Case Management Utilization

Bivariate logistic and subsequent multivariate logistic regression analyses were conducted to determine factors associated with integrated community case management utilization.

During bivariate analysis, sociodemographic characteristics such as the age of a child, sex of a child, caretakers age, religious affiliation, marital status, education status, occupation, and monthly income were found associated with ICCM utilization. Caretakers' awareness and perception of child's illness such as symptoms of illness, perception of severity of the problem, caretakers' symptoms perception as severe, awareness of danger signs, perceived danger signs, women health development army training status, certification of model families, and awareness ICCM service were also showed association with ICCM utilization. Finally, factors related to health facilities and community health workers include availability of health post, assignment of HEWs to the health post, the residence of HEWs proximity to the health post, availability of health center in the kebele, the timing of the visit to a health center and availability of transportation to the health center were also associated with ICCM utilization during bivariate analysis. Nevertheless, only six variables were found to be associated with integrated community case management utilization during the multivariate analysis.

Caretakers of children between 24 and 36 months were found 12.6% higher odds of utilizing ICCM service than the other age groups, (AOR = 1.26, 95%CI: 0.23, 0.90). Caretakers who had women's health developmental army training were five times the odds of utilizing ICCM service than those not trained, (AOR = 5.76, 95%CI: 3.57,9.30). Certified family model caretakers were 4 times the likely odds of utilizing ICCM service, (AOR = 3.98, 95%CI: 2.45, 6.46). Also, caretakers who perceived their child's health problem as severe were at the highest stake to utilize the ICCM service, (AOR = 5.29, 95%CI: 2.64, 10.60). Similarly, caretakers who know danger signs, (AOR = 2.76, 95%CI: 1.69, 4.50); and caretakers who had awareness of ICCM, (AOR = 5.42, 95%CI: 1.67, 17.58) were found to seek care at a health facility. Table 4 presents bivariate and multivariate regression analysis with study variables.

**Table 4:** Caretakers related factors associated with Integrated community case management (ICCM) utilization: a result from binary and multivariate logistic regression analysis in Dandi woreda, West Shewa, Ethiopia, 2022.

**Participant characteristics**		**Crude Odds Ratio (COR)**		**Adjusted Odds Ratio (AOR)**	
		**95% CI**	***p*-value**	**95%CI**	***p*-Value**
Care takers category:	Mother	1.18 (0.72, 1.94)	0.53		
	Non-mother	1	0.81		
Age of care takers:	15-24	1	0.66		
	25-34	1.17 (0.78, 1.76)	0.28		
	35-44	1.18 (0.73,1.91)	0.44		
	> 45	1.75 (0.60, 5.10)	0.47		
Sex of the child:	Male	0.87 (0.63, 1.19)	0.33		
	Female	1			
Age of child (in months)	<11 months	1.02 0 (0.62, 1.69)	0.94	1	0.97
	12–23 months	0.98 (0.62, 1.55)	0.44	0.84 (0.45, 1.59)	0.79
	24–35 months	1.65 (1.04, 1.62)	0.033	1.26 (0.23 0.90)	0.041
	36–59 months	1	0.21	0.88 (0.41, 1.89)	0.37
Marital status	Widowed	1	0.02	1	
	Not married	4.58 (1.52,13.87)	0.007	0.35 (0.06, 2.05)	0.25
	Married	2.11 (1.16, 3.85)	0.015	0.92 (0.33, 2.54)	0.87
	Divorced	1.53 (0.67, 3.49)	0.31	0.40 (0.11, 1.47)	0.17
Symptoms:	Diarrhea/vomiting /vomiting	1	0.20		
	Fever	1.29 (0.85, 1.98)	0.12		
	Cough	1.35 (0.91, 2.02)	0.85		
Marital status:	Widowed	1	0.02		
	Not married	4.58 (1.52, 13.87)	0.07		
	Married	2.11 (1.16, 3.85)	0.15		
	Divorced	1.53 (0.67, 3.49)	0.31		
Care givers income:	500 ETB & less	1	0.22		
	501-1000 ETB	0.42 (0.11, 1.64)	0.21		
	5001-1000 ETB	0.29 (0.07, 1.18)	0.08		
	501-1000 ETB	0.48 (0.09, 2.42)	0.37		
Education level	Not attended	2.51 (0.76,8.31)	0.13		
	Primary education	2.03 (0.61,6.78)	0.25		
	Secondary school & above	1.18 (0.34, 4.17)	0.79		
	Certificate and above	1	1		
Occupation:	Housewife	1	0.24		
	Merchant	1.18 (0.78, 0.77)	0.16		
	Employed	4.56 (0.53, 9.35)	0.22		
	Daily laborer	0.64 (0.37, 1.10)	0.11		
WHDA training:	No	1	0.000	1	
	Yes	6.8 (4.80, 9.73)	0.000	5.76 (3.57, 9.30)	<0.0001
Certified model family:	No	1		1	
	Yes	4.30 (3.05, 6.075)	0.000	3.98 (2.45, 6.46)	<0.0001
Severity:	Not sever	1		1	
	Moderate	0.92 (0.60, 1.43)	0.063	1.26 (0.69, 2.31)	0.71
	Sever	4.30 (2.61, 7.09)	0.004	5.29 (2.64, 10.60)	0.001
Awareness of danger:	No	1		1	
	Yes	4.70 (3.35, 6.59)	0.0078	2.76 (1.69,4.50)	0.03
Awareness of ICCM:	No	1		1	
	Yes	8.00 (5.58, 11.43)	0.000	5.42 (1.67, 17.58)	0.007
Assigned HEWs	Yes	1			
	No	2.43 (0.32, 3.4)	0.79		
Place of HEWs live	In the kebele	4.01 (4.52,9.93)	0.11		
	Out of the kebele	1			
Availability of transport:	No	1			
	Yes	6.12 (7.67,34.4)	0.032	3.9 (11.32, 26.75)	0.17
Place care sought:	Health post	0.39 (0.08, 3.45)	0.72	0.78 (0.02, 4.53)	
	Health center	9.12 (0.84, 12.32)	0.29	12 (0.12, 19.82)	
	Hospital	10.29 (0.960, 4.43)	0.91	4.0 (0.89, 23.36)	
	Private clinic	4.30 (9.11, 17.19)	0.20	1.10 (21, 34.49)	
	Others (traditional healers, drug venders, religious Center)	1.2 (0.15, 7.47)	0.12	0.2 (0.89, 25.07)	
	Not sought at all	1			

*p < 0.05 for both binary logistic and multiple logistic regression analysis*.

## Discussion

The study aimed at assessing the utilization status and associated factors of integrated community case management among caretakers of sick children <5 years of age in Dandi woreda, West Shewa zone, Ethiopia. Our study revealed the integrated community case management utilization status is widely reported across the woreda. About 57% of caretakers utilized ICCM service. The finding of this study is higher than other studies conducted in Ethiopia. For instance the service coverage in Boloso Sore Woreda, Wolaita zone is 25.3% (17), 22.5% in Hadiya zone (20), and 10.6% in Kindo Didaye district, Wolaita zone, Southern Ethiopia (21). The possible reason for the higher utilization of ICCM service in the current study includes the government effort to scale the program at the grassroot level communities through the health extension program. The higher coverage of the service is evident to reduce the common childhood illness such as diarrheal disease, pneumonia, and malaria.

In this study, caretakers of children between 24 and 36 months old were found to utilize ICCM service more than the other age groups. This finding is similar to the study in Kindo Didaye district, Wolaita zone, southern Ethiopia, where children between the age of 24 and 36 months were found to utilize the ICCM service the most (20). The possible reason for the caretakers to seek ICCM service utilization for these age groups include the child's tendency to report sickness and the caretakers' observation of the child's health status.

It was found that caretakers trained in the women's health development army were found six times more likely to seek care for their sick child in comparison to the other groups (20, 22, 23). The finding of this study corresponds to the study conducted by Yohannes et al. (20), which that showed members of the women's development army were more likely to utilize ICCM services. Being a member of the women's health development army means a woman who knows the packages of a health extension p and practices them all resulting in a decrease in child mortality and morbidity through enhanced integrated community case management utilization (23). The finding implies that the women's development army is important in raising the behavior of the community to use ICCM service for the sick child. This was well-documented in the cross-sectional study conducted in the four regions of Ethiopia (24).

Our study also revealed that being a certified model family is associated with seeking ICCM for their sick child. A certified model family household is a household that graduated from the health extension program after fulfilling health extension packages and has adequate knowledge and practice of integrated community case management for sick children. These groups of the community are trained in health extension packages and implementing these packages after the training, have knowledge of child health care, and can influence their neighbors to adopt the same practices (25). The health extension package is one means of implementing the sustainable development goal (SDG) by bringing main maternal, neonatal, and child health interventions to the community (26). Evidence has shown that there is a variation between model families and non-model families regarding the utilization of family health services including IMNCI and model families utilizes the most (27).

Caretakers' perception of a child's illness and knowledge of danger signs were found to be associated with the utilization of integrated community case management. Caretakers who perceived their child's health problem as severe were reported to utilize the ICCM service 5 times more likely in comparison to those who were unaware. It was reported that when caretakers observe the danger signs such as fever and fast breathing, they were more likely to visit health facilities and utilize ICCM. Congruently, studies conducted in Southern Ethiopia (17), Dawro Zone, South-west Ethiopia (28), Oromia, Ethiopia (29), Kenya (30), and India (31) have revealed that the mother's/caregiver's perception of the high severity of illness is associated with of integrated community case management service.

Caretakers who had awareness of ICCM were also found to seek care for their sick children more than those not. This corresponds to the result of the study conducted by Serawit Samuel and Aseb Arba (17), Shaw et al. (29). When caretakers are aware of a child's health condition and if the condition is deteriorating, the caretakers would likely visit the health facilities for ICCM service. Knowledge of ICCM service helps to recognize the clinical symptoms that necessitate immediate action and appropriate management of disease conditions. In doing so, they play a big role in reducing/preventing morbidly and mortality of the children.

### Limitations of the Study

The study included caretakers whose child was sick in the last 3 months as such there might be a recall bias.

## Conclusion

The utilization of the integrated community case management in the current study area is high, about 57% of caretakers for children under 5 years old utilized ICCM service. The study revealed that age of the child, mothers/caretakers' awareness of ICCM, danger signs, perception of the illness severity, training on women's health developmental army, and graduation as a model family were associated with integrated community care utilization in Dandi Woreda, West Shewa, Ethiopia. Therefore, it is recommended that health workers at each level promote health education using community-level health developmental army training focusing on creating awareness on the areas of common childhood illness symptoms, severity, danger signs of a sick child, and care-seeking behavior. Educating mothers/caretakers and the community on the importance of ICCM service mainly for children under 5 years old were necessary, including enhancing women's development army training and empowering health extension workers to continually train and certify families to be “a model” in community-based healthcare packages. Moreover, improving public–private partnerships in the areas of ICCM service delivery and addressing the communities through behavioral change communication on ICCM service demand creation and utilization are also highly recommended. The result of the study is highly significant in contributing to reducing child morbidity and mortality by encouraging policymakers, health practitioners, and local health administrators by strengthening and/or redesigning resource-efficient and effective community-based strategies that are highly significant to least-income countries such as Ethiopia.

## Data Availability Statement

The raw data supporting the conclusions of this article will be made available by the authors, without undue reservation.

## Ethics Statement

The studies involving human participants were reviewed and approved by Wollega University Institutional Review Board. The patients/participants provided their written informed consent to participate in this study.

## Author Contributions

LD developed the proposal, analysis, write-up, manuscript development, and review. FN analyzed the data and edited and reviewed the manuscript. Both authors contributed to the article and approved the submitted version.

## Conflict of Interest

The authors declare that the research was conducted in the absence of any commercial or financial relationships that could be construed as a potential conflict of interest. The reviewer AB declared a shared affiliation with the author LD to the handling editor at the time of review.

## Publisher's Note

All claims expressed in this article are solely those of the authors and do not necessarily represent those of their affiliated organizations, or those of the publisher, the editors and the reviewers. Any product that may be evaluated in this article, or claim that may be made by its manufacturer, is not guaranteed or endorsed by the publisher.
